# First person – Mugagga Kalyesubula and Ramgopal Mopuri

**DOI:** 10.1242/dmm.048941

**Published:** 2021-03-18

## Abstract

First Person is a series of interviews with the first authors of a selection of papers published in Disease Models & Mechanisms, helping early-career researchers promote themselves alongside their papers. Mugagga Kalyesubula and Ramgopal Mopuri are co-first authors on ‘High-dose vitamin B1 therapy prevents the development of experimental fatty liver driven by overnutrition’, published in DMM. Mugagga is a PhD student in the lab of Dr Hay Dvir at the Volcani Center – Agricultural Research Organization (ARO), Rishon LeZion, Israel and The Hebrew University of Jerusalem, Rehovot, Israel, investigating therapy development for chronic metabolic diseases such as non-alcoholic fatty liver disease. Ramgopal is a postdoctoral researcher in the lab of Dr Hay Dvir at the Volcani Center – ARO in Rishon LeZion, Israel, developing therapeutic approaches for the management of fatty liver diseases.


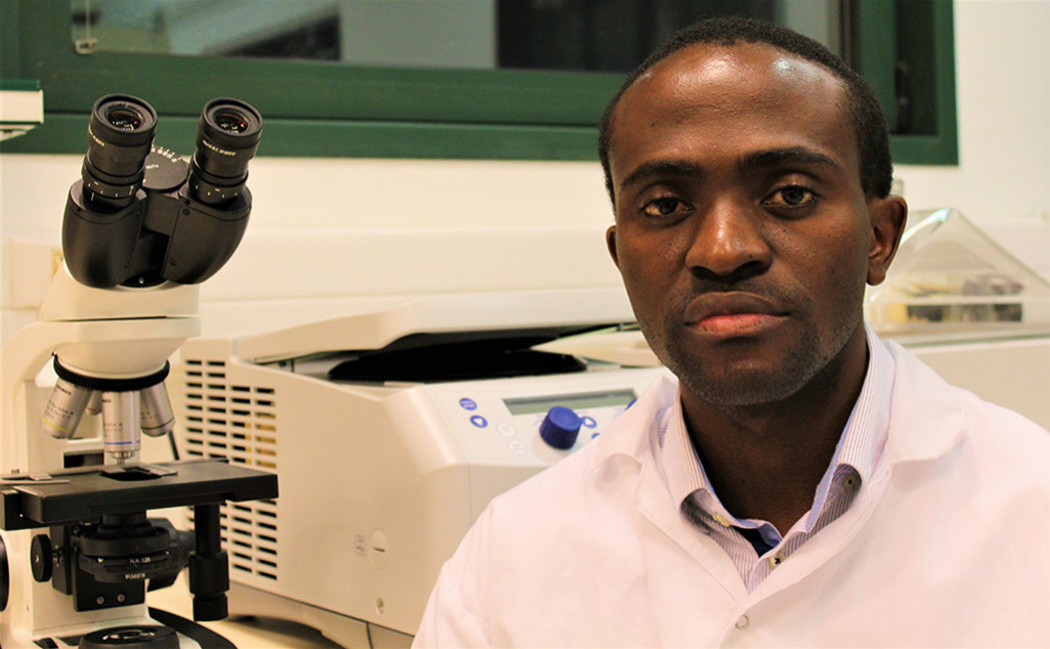


**Mugagga Kalyesubula**


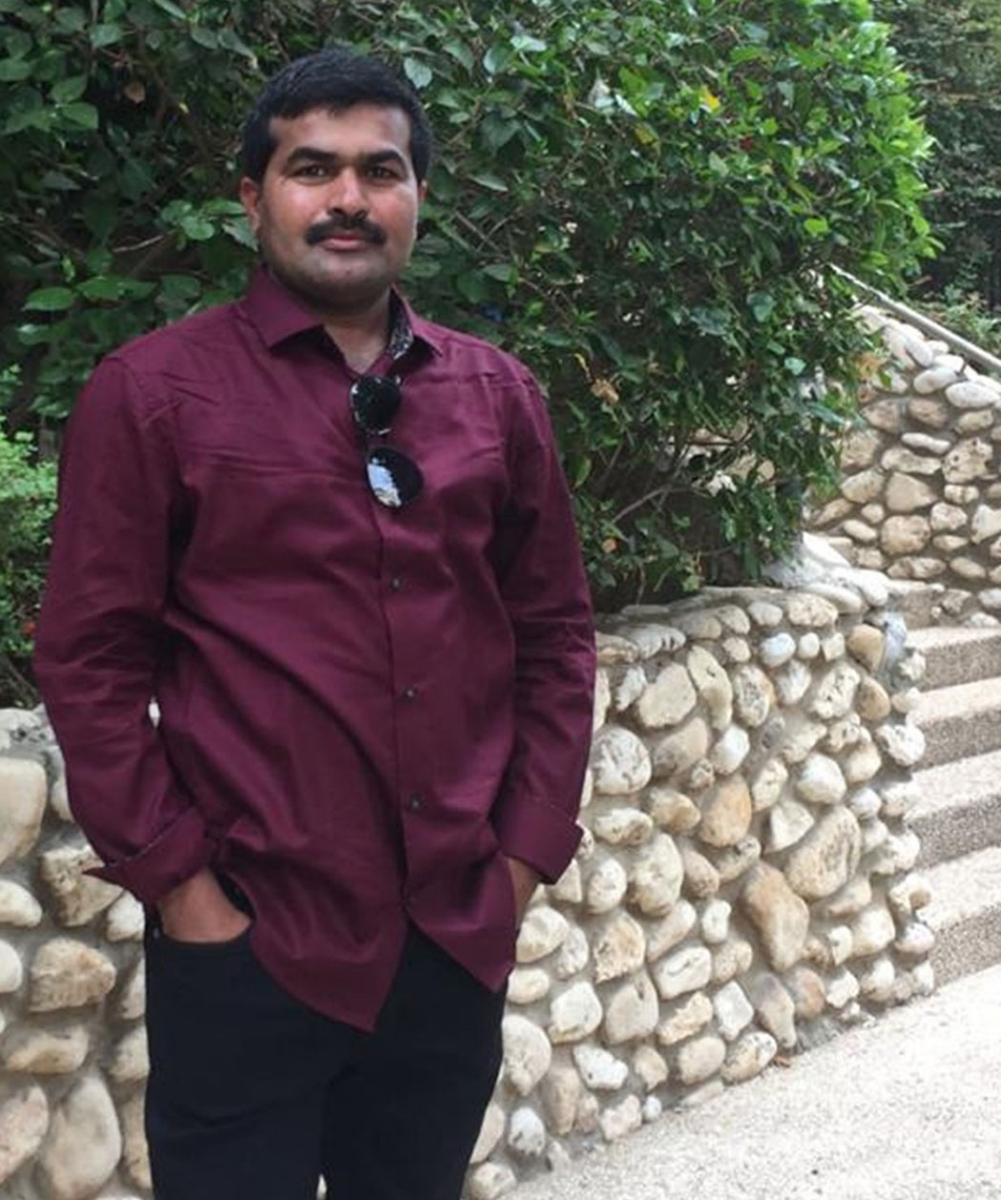


**Ramgopal Mopuri**

“There is a global increase in the prevalence of nonalcoholic fatty liver disease.”

**How would you explain the main findings of your paper to non-scientific family and friends?**

**MK+RM:** There is a global increase in the prevalence of non-alcoholic fatty liver disease (NAFLD) due to excess caloric intake and a sedentary lifestyle, currently affecting ∼25% of the global population. Despite the intensive research to uncover the pathogenesis of NAFLD, it is still incompletely understood. Animal models are crucial in filling this gap. Our research revealed that we could employ sheep as a large-animal model for studying fatty liver since they develop hepatic steatosis along with key metabolic comorbidities, such as hyperglycemia, hyperinsulinemia and insulin resistance, due to overnutrion. Moreover, there is currently no FDA-approved drug for treating NAFLD. Our findings show that high-dose vitamin B1 therapy substantially prevent nutrition-induced fatty liver in sheep, despite being overnourished. Vitamin B1 and our large-animal model for metabolic fatty liver could play a part in overcoming this global epidemic.

**What are the potential implications of these results for your field of research?**

**MK:** The finding that vitamin B1 – a safe over-the-counter substance – prevents nutrition-induced fatty liver may be important to the drug discovery efforts to treat NAFLD. We have also shown that we can employ sheep as a large-animal model, hence expanding the spectrum of animal models that can be utilized in pathogenesis and therapy investigations.

**RM:** In our paper, we demonstrate a profound antisteatotic ability for vitamin B1 in sheep. If translated to humans, such results may be of substantial clinical value for the management of fatty liver-related disorders.

**What are the main advantages and drawbacks of the model system you have used as it relates to the disease you are investigating?**

**MK:** Our sheep model of fatty liver is based on overnutrition, which is the main factor associated with NAFLD in humans. The weight of our breed of sheep is equivalent to that of humans and, therefore, increases the translation potential. From a research perspective, this large-animal model enables collection of considerable quantities of biological specimens to perform multiple biochemical, histopathological and molecular analyses. However, working with large animals such as sheep is labor intensive.

**RM:** There is great interest in large-animal models to study different aspects of human diseases, allowing critical understating of disease mechanisms and the opportunity to investigate potential therapies to increase the success in clinical trials. Our current and also previous studies (Kalyesubula et al., 2020a) show that sheep can be employed to study fatty liver and related metabolic morbidities, such as insulin resistance. One drawback of this large-animal model has to do with its different digestive system, which compromises its suitability to test oral treatments.

**Figure:**
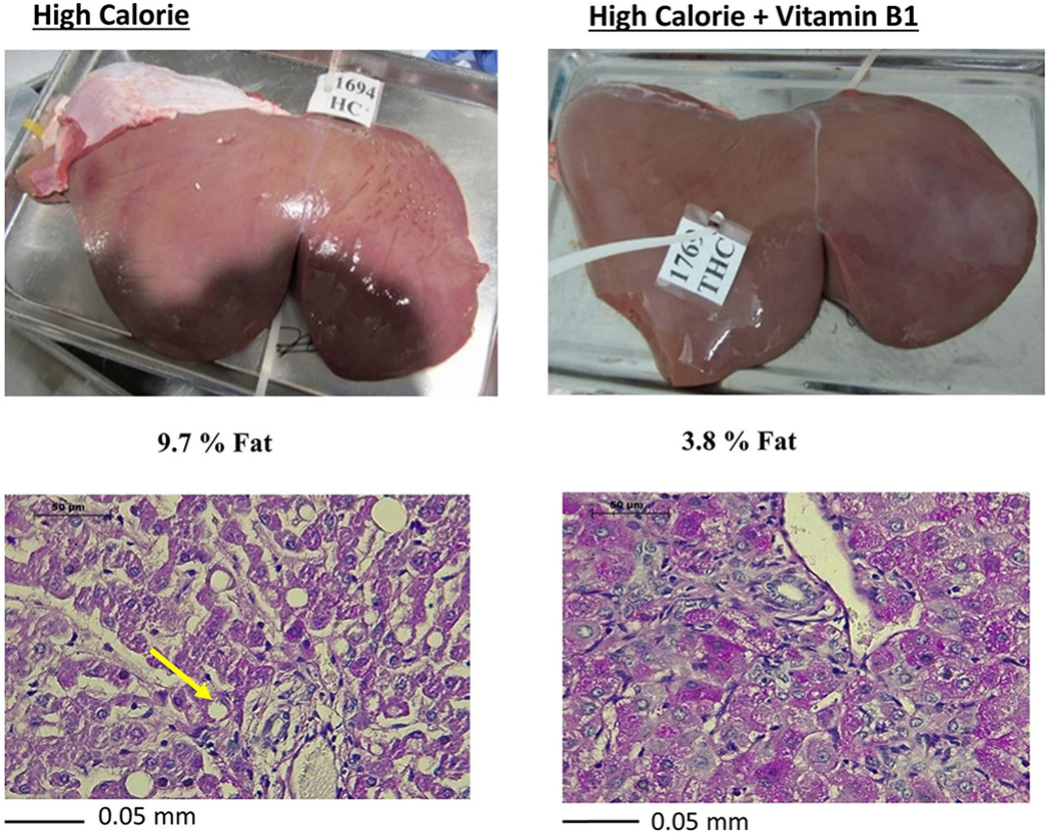
**Gross and corresponding histopathological images showing that vitamin B1 treatment prevents nutrition-induced hepatic steatosis.**

**What has surprised you the most while conducting your research?**

**MK:** It was the finding that vitamin B1 lowered the fat content of livers of animals under a high-calorie diet to a level similar to that of livers of animals under a low-calorie diet. At the level of gene expression, vitamin B1 effects were detected on hepatic lipid-droplet formation and lipidation of very-low-density lipoprotein, implying that its effects on lipids metabolism may not only be mediated through its classic coenzyme roles.

**RM:** I was surprised by the remarkable response of sheep to subcutaneous administration of an essential vitamin in preventing hepatic steatosis.

**Describe what you think is the most significant challenge impacting your research at this time and how will this be addressed over the next 10 years?**

**MK:** A major challenge in tackling NAFLD is the need to address its multifaceted nature. This has limited the development of effective interventions, and emphasizes the need for multiple robust animal models as well as the need for therapies combining more than one drug. Our large-animal model, as demonstrated in this study, has the potential to play an important part in NAFLD research. More studies are warranted to translate therapeutic candidates, such as vitamin B1, into clinical studies and, eventually, into clinical practice for management of the NAFLD epidemic.

**RM:** Owing to its vital role in energy metabolism, we explored the therapeutic potential of vitamin B1. Excitingly, the substantial effect of vitamin B1 on reducing hepatic steatosis seems very promising. Since vitamin B1 did not reduce the whole-body insulin resistance, it would be of great interest to evaluate the efficacy of combination therapies, i.e. of vitamin B1 and insulin sensitizers.

“[…] vitamin B1 – a safe over-the-counter substance – prevents nutrition-induced fatty liver […]”

**What changes do you think could improve the professional lives of early-career scientists?**

**MK:** Improving means collaborations and networking, to learn from other scientists both in my field and in other fields. This year has been challenging as a PhD student because of the limited travel due to the COVID-19 pandemic and, therefore, we could not meet with other researchers at international conferences. Hopefully, a mechanism can be put in place to compensate for the lost opportunities.

**RM:** Establishing career mentoring programs geared at helping early-career scientists to gain self–confidence and inspire them to carry out independent research activities would highly improve their professional lives.

**What's next for you?**

**MK:** I would like to learn more about the molecular mechanisms of chronic metabolic disease development, such as NAFLD, and how to develop interventions to address them. Currently, I am planning to finish my PhD thesis and move on to a postdoctorate.

**RM:** After obtaining encouraging results about the potential benefits of vitamin B1 in addressing nutrition-induced fatty liver, the next step would be testing it in other monogastric animal models and, eventually, bringing it to clinical trials in humans.
